# Case of Difficult Diagnosis

**Published:** 1861-02

**Authors:** D. C. O’Keefe

**Affiliations:** Atlanta, Georgia


### ARTICLE II.

### Atlanta Medical Society.

*Case of Difficult Diagnosis.* By D. C. O'KEEFE, M. D., Atlanta, Georgia.

Mr. H., aged 63, a carpenter by trade, tall and spare make, has had slight dyspeptic symptoms most of his life-time, from which, however, he has suffered no great amount of inconvenience. He was in the enjoyment of usual health until June, 1859, at which time he suffered for two or three weeks from slight dyspnœa, loss of strength and moderate œdema of feet and ankles. Under an anti-dropsical course of treatment he regained his wonted health, which he enjoyed until March, 1860. At this time his dyspnœa, debility and œdema returned—the latter slightly and irregularly—and continued to increase gradually, the dyspnœa being induced or aggravated by exertion of any kind, even talking, until the 20th of December last, when I visited him.

Up to this time, he was "up and about," but had now taken his bed. He lay usually on his back, and could not lie on either side without an aggravation of his dyspnœa. The skin was dry and harsh, but natural in temperature; tongue had a very slight whitish fur on its posterior half; considerable tenderness on pressure in epigastric region, which did not extend to the right or left. The urine was highly colored, and passed in normal quantities; bowels rather torpid, appetite indifferent, sleep interrupted, pulse hard and strong, numbering about 100; feet very slightly œdematous, if at all—no pain.

The dyspnœa was the prominent symptom at this time; the respiratory function was peculiarly disturbed; there was slight cough, or rather a hawking or frequent clearing of the throat and dislodgment of tough phlegm. There seemed to be no obstruction to the free ingress of air, and yet he was frequently making deep inspirations to inflate the lungs.—He felt, he said, as if he could not get air enough, and these deep inspiratory efforts were to supply that want. In the intervals of the deep inspirations, the respiratory movements were frequent and imperfect—in familiar language, "he panted for breath."

He felt, he said, as if his right lung had shrunk away and was not larger than his hand; at other times he would place his hand across his sternum and say there was a stricture within his chest at that place. Nothing worth mentioning was elicited by a physical examination of the thorax; the pulmonic resonance was normal everywhere except that cardiac dullness existed over more space than natural—no cardiac murmur discernible. The lungs were fully expanded only by the deep and labored inspirations; in the short and hurried efforts, the air entered only about the superior half of each lung. The difficulty *seemed* to consist in the want of power to perform a full and perfect respiratory act; but whence originated this loss of power?

In consultation with a medical friend—Professor H. W. Brown—it was determined that his difficulties had their origin in chronic inflammation of the stomach, small bowels and perhaps liver, and that the respiratory embarrassment depended on general failure of the nervous energies of the system. Treatment. Minute doses of Calomel with Bismuth, Quinine and anti-spasmodics, and a blister to epigastrium. He received no benefit from this treatment—indeed the dyspnœa increased to such a degree that he could not assume the horizontal position, nor procure any sleep. He sat in his chair for many days and nights together without sleep, except a momentary nap. A singular feature became developed at this time in connection with the efforts to obtain sleep. As soon as he dozed off to sleep, the function of respiration would entirely cease, and in half a minute he would wake up in great distress for breath. While in this condition, he obtained several hours' sleep on two or three occasions from the use of Morphia.

In the meantime, the lower extremities, from the hips down, became anasarcous to a considerable extent—indeed, I think there was more or less general anasarca. He was briskly purged with salines several times during the couple of weeks of which the above is a record, with no decided improvement. Some alcoholic stimulants were also used with apparent aggravation of the symptoms.

In this condition, having occasion to leave home for a few days, I advised him to call another physician, which he did. When this physician saw him, the dropsical condition of the extremities had increased, and the dyspnœa was no better. He prescribed for him a powder composed of calomel, nit. potass, pulv. squills and digitalis every four hours. In the course of three or four days a *decided* diuretic and moderate purgative effect ensued, followed by almost entire exemption from dyspnœa and disappearance of anasarca, except in the feet and ankles. His appetite was much better, could sleep without any difficulty, and, in all respects, his condition was decidedly improved. After about a week's continuance of the treatment, slight ptyalism was induced, and the powders were discontinued. He is now taking the syrup of Iodide of Iron, and is still on gaining ground.

*Remarks.*—I have headed these notes "Difficult Diagnosis." It is a name not yet introduced into our nosological classifications, and yet it is no less true that the cause of science would be frequently advanced by an honest confession of our ignorance. It is better for the cause of truth and humanity to say, "I cannot diagnose this case," and treat symptoms as they arise, than to form an opinion and base a treatment on inadequate data.

What was the pathological condition which gave origin to the symptoms herein recorded? I confess I am unable to decide with any degree of satisfaction. Dyspnœa, in the beginning, was the most prominent symptom, but on this *alone* we could predicate nothing; it is an expression of so many pathological states essentially differing one from the other, that we cannot rely on it alone for a correct solution of the case. I will briefly refer to some of the diseases of which embarrassed respiration is a symptom to show that, by the facts in the record, they must be excluded from our consideration. It was not hydro-thorax, nor pleurisy with effusion, because of the absence of the well-marked physical signs which attend them. It was not pulmonary emphysema for the same reason; neither was it pneumonia, phthisis, nor capillary bronchitis, because its history, general symptoms and physical signs do not belong to either of these diseases. Was it connected with a cardiac affection? On this point, the only evidence we have was an increased dullness in the cardiac region—there was no appreciable change in the heart sounds. But might there not have existed hypertrophy with dilation of one or both of the cavities of the heart, thereby inducing embarrassment of the pulmonary, as well as the general circulation? In this way an effusion into the pulmonary tissue would occur, denominated by Læennec pulmonary œdema, and the embarrassed respiration would be the consequence; this supposition would also explain the general anasarca. Unfortunately for this hypothesis, it is not sustained by the physical signs. In the first place, in nineteen cases out of twenty, cardiac hypertrophy with dilation is the result of mechanical obstruction of the orifices of the heart; but the record says there was no appreciable change in the cardiac sounds. And secondly, in œdema of the lungs, we have feeble respiration and the crepitant rale, neither of which existed. Why was not the original diagnosis correct? The result of treatment, often of great value in doubtful cases, tends to prove, in my opinion, that the dyspnœa was dependent on dropsical collection somewhere. On the whole, I prefer to regard it a case of "doubtful diagnosis."

